# Origins of the Schottky Barrier to a 2DHG in a Au/Ni/GaN/AlGaN/GaN Heterostructure

**DOI:** 10.1021/acsaelm.2c01138

**Published:** 2022-09-21

**Authors:** Huy-Binh Do, Jinggui Zhou, Maria Merlyne De Souza

**Affiliations:** † Department of Electronic and Electrical Engineering, University of Sheffield, Mappin Street, Sheffield S37HQ, U.K.

**Keywords:** p-type contact to GaN, Ni/Au, Schottky contact, 2DHG, thermionic field emission, Mg traps in u-GaN

## Abstract

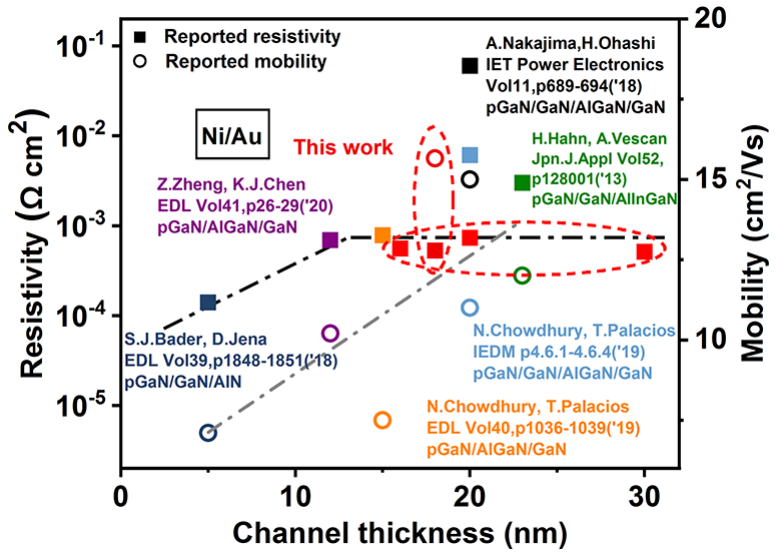

We report the influence of thickness of an undoped GaN (u-GaN) layer on current transport to a 2DHG through the metal/p++GaN contact in a GaN/AlGaN/GaN heterostructure. The current is dominated by an internal potential barrier of 0.2–0.27 eV at the p+ GaN/u-GaN, which increases with thickness from 5 to 15 nm and remains constant thereafter due to Fermi pinning by a defect at ∼0.6 eV from the top valence band. We also report a nonideality factor, *n*, between 6 and 12, for the combined tunneling current through the p+GaN/u-GaN to the 2DHG. Our contact resistivity of 5.3 × 10^–4^ Ω cm^2^ and hole mobility, μ, of ∼15.65 cm^2^/V s are the best-in-class for this metal stack on a GaN/AlGaN/GaN heterostructure, reported to date.

Establishing a low resistivity contact to a two-dimensional hole gas (2DHG) still remains a formidable challenge, despite recent advances in e-mode p-channel transistors in GaN. These are desirable for monolithic integration of beam forming with front-end modules in 5G antenna arrays and gate drivers of GaN power devices. Although heterogeneous integration of a p-type silicon MOSFET with an n-channel GaN HEMT has been demonstrated, the technology suffers from challenges of heat dissipation.^1,2^ The dream of realizing CMOS in GaN has been spurred by demonstration of scaled p-channel FINFETs^3,4^ to overcome trade-offs associated with on/off ratio, threshold voltage, and the maximum on-current, affected primarily by the poor mobility of holes, in p-channel FETs.^5^ Despite on-currents of 66 and 140 mA/mm in comparison to 1.6 mA/mm in ref (6), there still remains considerable scope for improvement.^7^

This work aims to develop an understanding of the inherently high contact resistance to a 2DHG in GaN. The high resistivity has been attributed partially to a poor efficiency of Mg activation, usually lower than 1%, even at a doping concentration of 1.0E20 cm^–3^, due to the high activation energy of ∼170 meV in Mg-doped MOCVD-GaN.^8,9^ Recent progress via low temperature molecular beam epitaxy (MBE) has demonstrated a reduction of donor-like defects that tend to compensate for the p-type doping, to levels of ∼1.0E17 cm^–3^, resulting in higher activation^10^ by a factor of 10. Regrown contacts in combination with high work-function metals such as Ni/Pd can help reduce the contact resistivity.^11^ Nevertheless, regrowth is not a desirable technique, and Au is preferred over Pd as a capping layer.

Beyond the metal/semiconductor contact, motivated by the desire for CMOS technology, there is a smaller body of work^11−17^ attempting p-channel devices, from which reported values of the resistivity of the contact to a 2DHG, and the corresponding hole mobility, are depicted in the figure in the abstract. In such devices, typically an undoped GaN (u-GaN) layer may lie between the contact and the 2DHG, as highlighted in Figure 1, resulting in a range of values of contact resistivity of Ni/Au from 4.9 × 10^–6^ Ω cm^2^, in a structure without a u-GaN layer (at an estimated Mg doping concentration of 3.0E19/cm^3^),^14^ to ∼1.0 × 10^–2^ Ω cm^2^ for a contact separated by 20 nm of GaN.^16^ Not all reported structures are based on GaN/AlGaN; Vescan et al. achieved 7.3 × 10^–4^ Ω cm^2^ for a GaN thickness of 3 nm on quaternary AlInGaN.^18^ On the other hand, Palacios et al. demonstrated ∼1.0 × 10^–2^ Ω cm^2^ for a GaN thickness of 20 nm.^16^ From these preceding articles, it is easily apparent that the introduction of an undoped layer affects the resistivity of the contact to the underlying 2DHG. With the exclusion of the data points arising from this work, the figure in the abstract is suggestive of a trade-off of the contact resistivity with mobility, linearly with thickness of the u-GaN layer. Our motivation, therefore, is to attempt physical insight into the origins of this behavior, because undoped GaN layers are essential in HEMTs to reduce scattering of carriers in the 2DHG. However, our previous work highlights reducing the on/off ratio in devices with thickness in excess of 16 nm due to loss of electrostatic control.^19^

**Figure 1:**
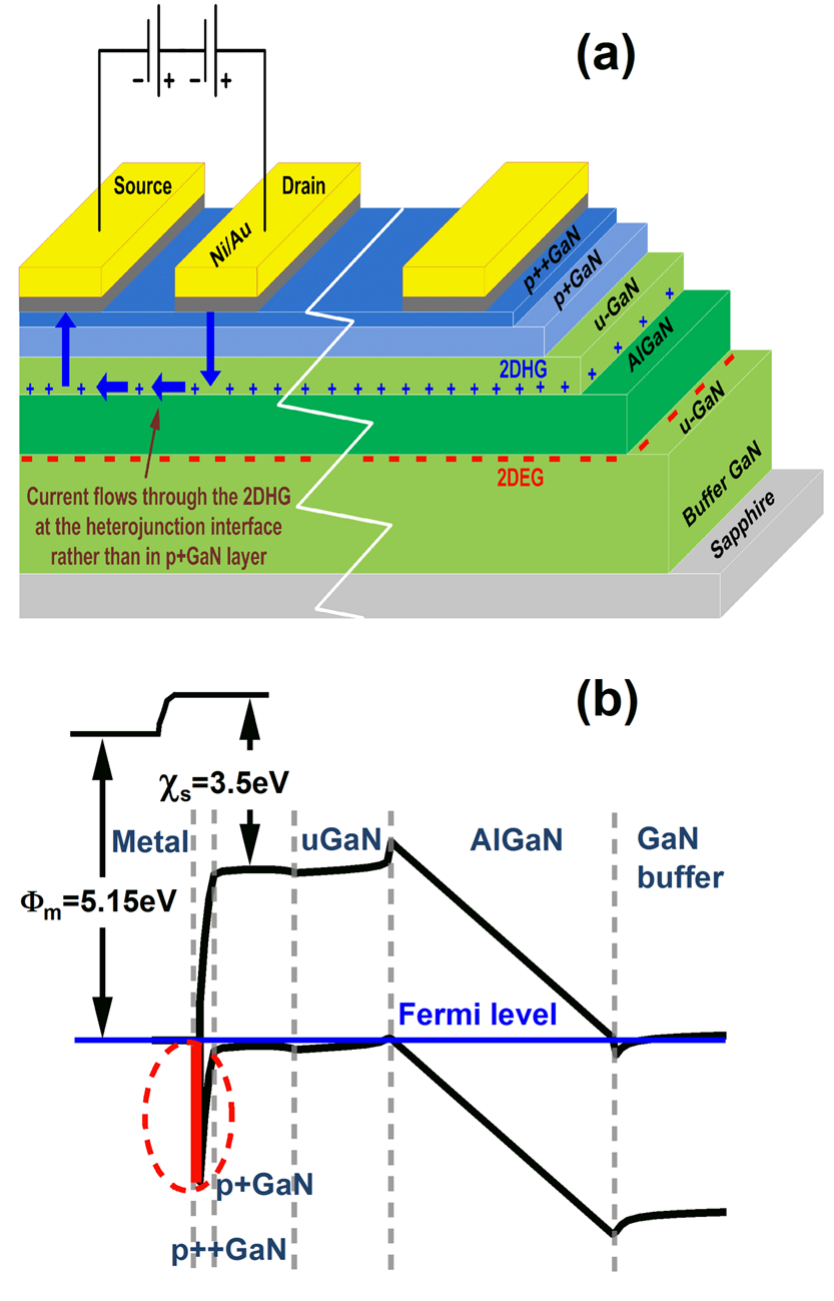
(a) Cross section of the substrate and TLM contacts in Ni/Au, highlighting the contributions of the total resistance to the current flow path. (b) The corresponding zero bias energy band diagram of the structure with the theoretical Schottky barrier of 1.65 eV, represented by eq 1, highlighted in red.

Our device (Supporting Information A), highlighted in Figure 1(a), includes a current path consisting of a maximum SB of 1.65 eV (Figure 1(b)) between the Ni/Au metal stack and a p++ GaN layer that yields an extracted value of contact resistivity ρ_c_, sheet resistance of the semiconductor regions consisting of p+ GaN and a u-doped semiconductor channel represented by *R*_c_, and the resistivity of the 2DHG(∝1/μ), as limited by the Hall mobility. A bending of *I*–*V* curves around 0 V obtained from TLM measurements of a sample with u-GaN thickness of 16 μm in Figure 2(a) indicates the existence of a Schottky barrier between the metal and the 2DHG. Our extracted transfer length, based on total resistance–distance characteristics shown in Figure 2(b), is typically 1.2 ± 0.19 μm (Supporting Information B), the fluctuation representing the spread of current with thickness of u-GaN. The sheet resistance (Supporting Information B) increases linearly with thickness between 34.7–42.4 kΩ/sq with a limiting value of 27.2 kΩ/sq extracted in Figure 2(c). Our results lie between those of Jena et al., who achieved 8.89 kΩ/sq, with Pd/Ni and p-InGaN contacts with 15 nm of u-GaN channel,^11^ and Chen et al., who reported 56 kΩ/sq for 12 nm AlGaN.^12^ Using p-InGaN is a major contributor to the reduction of the contact resistivity because the conduction band offset between GaN and InN is 1.6 eV,^20^ leading to an electron affinity of InN of 5.91 eV, higher than any metal work-function. The ternary compound of p-GaN and InN has an estimated valence band offset of 1.15 eV^20^ that should reduce the resistance between metal and 2DHG significantly. The values of resistivity obtained in this study vary from ρ_c_ ≈ 5.6 × 10^–4^ Ω cm^2^ (16 nm) to ρ_c_ ≈ 5.1 × 10^–4^ Ω cm^2^ (30 nm) and show a relative independence to thickness of u-GaN, in Figure 2(c), contrary to the reported trend from the figure in the abstract. Figure 2(d) shows the Hall mobility with u-GaN thickness measured using Van der Pauw structures with an average value of 15 cm^2^/V s, similar to that achieved by AIST.^15,21^ It is noted that Hall mobility and sheet hole density are determined by
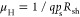
1where μ_H_ is the Hall mobility, *p*_s_ is the sheet hole density, and *R*_sh_ is the sheet resistance. This equation explains why the sheet hole density and mobility track each other oppositely (Figure 2(d)), when extracted via this method with a relative immunity to thickness of u-GaN (between 16 and 30 nm). The sheet hole density at the GaN/AlGaN interface can be confirmed by using *C*–*V* characteristics.^22^ To study current transport through the contact, temperature dependent *I*–*V*s at a gap length of 5 nm, for an 18 nm thick u-GaN, are reported in (Figure S1). The SB, Φ_B_, can be extracted from a semilog plot of *I*–*V* as depicted in Figure 3(a) as
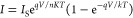
2where *I*_S_ = *AA* * *T*^2^e^–*qΦ*_B_/*kT*^; *n*, the diode ideality factor, is obtained from the slope; and the SB, Φ_B_, can be obtained from the intercept, *I*_s_, as
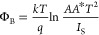
3

**Figure 2:**
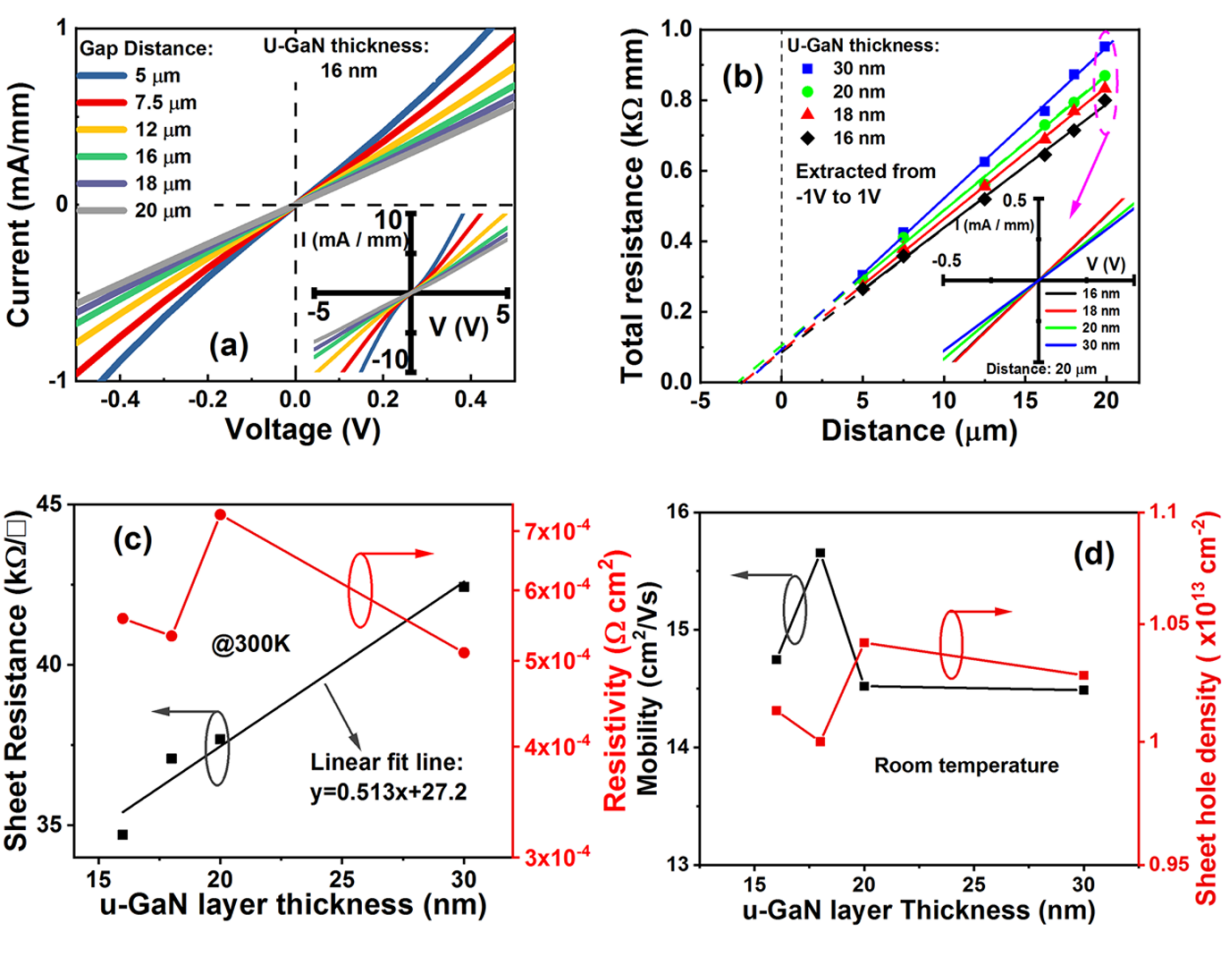
(a) *I*–*V* characteristics as a function of spacing of contacts for a u-GaN thickness of 16 nm measured at 300 K. (b) Total resistance measured at 300 K versus gap distance of the TLM structure as a function of u-GaN thickness. The total resistance increases with the thickness of the GaN layer. (c) Sheet resistance and contact resistivity as a function of u-GaN thickness show a linear increase of the former and independence of the latter with thickness. (d) Extracted mobility and sheet hole density versus u-GaN thickness indicate a marginal peak at ∼18 nm within the error margins of variation from sample to sample.

**Figure 3:**
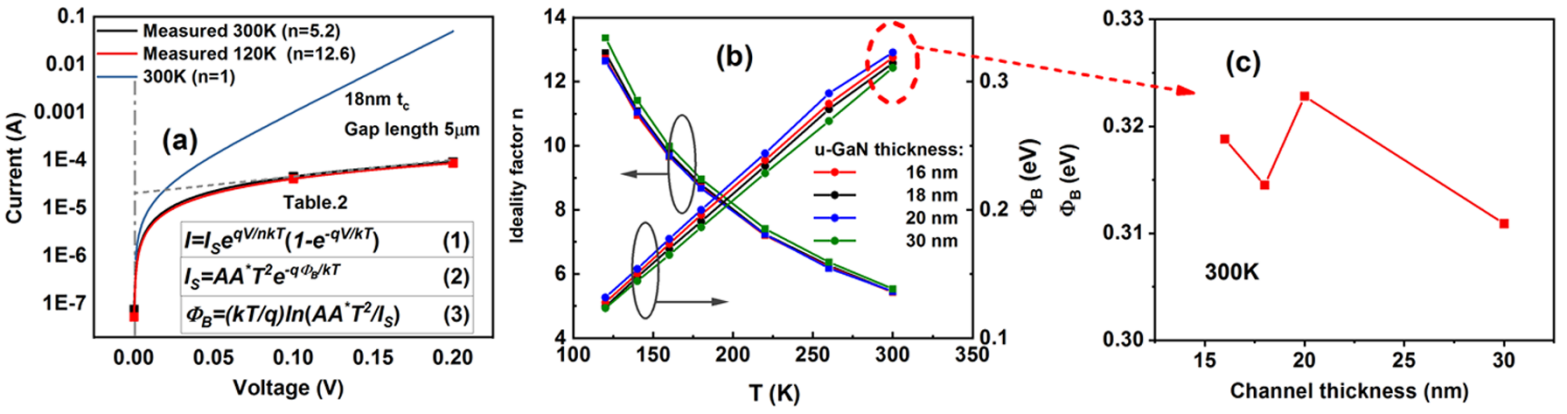
(a) The method of extraction of the nonideality factor *n* from the current–voltage characteristics in the diode region at 120 and 300 K. In the Schottky region, current is almost independent of temperature; therefore, *n* changes (proportional to *T*). *n* is obtained from the slope: *q*/slope · *kT*; Φ_B_, is obtained from the intercept, *I*_s_. A hypothetical ideal curve for *n* = 1 at 300 K is indicated. (b) Plot of the ideality factor *n* and Schottky barrier Φ_B_ of a Ni/Au contact as a function of temperature and thickness of the u-GaN layer. (c) Schottky barrier versus u-GaN thickness at 300 K corresponding to the data in b.

The Richardson constant A*, defined by A* = 4*πqk*^2^m*/*h*^3^, is traditionally obtained from a plot of ln(*I*/*T*^2^) versus (1/*T*). Based on an effective mass of 0.16, we obtain a theoretical Richardson’s constant of 19.2 (Supporting Information D) and note that an error of 2 in A* results in an error of only 0.7*kT*/*q* in Φ_B_.^23−28^ From Figure 3(a), it is observed that the *I*–*V* characteristics plotted on a log–linear scale have significant nonlinearity, resulting in a temperature dependent ideality factor between 6–12 that we report here for the first time in this type of contact to a 2DHG in GaN. Figure 3(a) also shows a hypothetical curve with *n* = 1, whereas *n* > 1 is evidenced by the flattening of the *I*–*V* characteristic.^29^ Near to 0 V, there is little change of current with voltage; hence, *n* = 6–12 reflects the variation only due to *T*, in the term (*nkT*) in eq 3. Physically, this signifies a contribution of tunneling to the current transport mechanism from a large number of defective states in the surface layers, which reduces the SB at lower temperature, due to an increase in field emission (Figure 3(b)). These defects might be assigned to the Ga vacancy that acts as acceptor (a result of removing native GaO_*x*_ on the GaN),^30^ with an energy level of 0.1–0.3 eV^31^ and 0.15 eV^32^ from the valence band maximum. This behavior is far from ideal thermionic field-emission theory used previously to extract the SBH to p-GaN,^7,25,33,27^ which assumes a single SB fitted to the entire range of temperature, from a plot of resistivity versus temperature, which is clearly not the case when *n* ≠ 1. Okumura reports three ranges of behavior for their contacts based on (i) *N*_A_–*N*_D_ < 2e19/cm^3^, resulting in the onset of Schottky behavior, (ii) Ohmic behavior between 3E19–7E19/cm^3^ attributed to hole tunneling via field emission, whereas (iii) higher doping concentrations show a peculiar increase in resistivity due to deactivation of Mg but without any accompanying Schottky behavior in their *I*–*V*.^10^ They propose this behavior to arise from tunneling through deep level defects and interfacial traps of compounds including accumulated Mg at the surface, via trap assisted tunneling, consistent with the high nonideality factors we observe.

In comparison to many other studies, there is no annealing involved in our process. We assume that theories related to the formation of a NiO interface^34^ or the dissociation of Mg–H complexes^35^ that prevent activation of Mg may well not apply to our case, with relatively thick Ni/Au layers. Our experiment indicates that the most likely reason for the quality of the Schottky contact is the thin amorphous layer present on the p-GaN surface, consisting of Ga_2_O_3_ and adsorbed carbon or hydrocarbon contamination formed during exposure to air of the GaN surface immediately after MOCVD growth.^36^ This layer is removed via wet chemical etching to improve the contact.

Figure 3(c) shows the SB extracted from the experimental *I*–*V* curves using eqs 2 and 3 to be largely invariant with thickness of the u-GaN layer larger than 15 nm, at ∼0.32 eV at 300 K, which does not explain the reported trend of thinner u-GaN layers resulting in lower contact resistivity (cf. figure in abstract). Although there could be room for marginal improvement of the reported barrier height, this figure proves that the resistivity of the contact metal stack is unrelated to the surface layer alone.

The influence of the u-GaN thickness is examined via TCAD simulations (Supporting Information E), by hypothetically varying its value from 2 to 30 nm with a constant ρ = 5.3 × 10^–4^ Ω cm^2^ in all simulations. The resultant *I*–*V* characteristics in Figure S4 (Supporting Information E) fit well with experiment at all channel thicknesses (16, 18, 20, and 30 nm). We include interface traps (*Q*_it_) of 1.0E17 cm^–3^, with the energy level *E*_v_ + 0.6 eV (Supporting Information E), believed to be carbon contaminants at the p+GaN/u-GaN interface to match the experiment.^37^ Depending on the deposition condition, carbon contamination has been reported previously to be 1.0E16 to 1.0E18 cm^–3^.^37^ The simulated *I*–*V*s show an increase of current with reducing channel thickness in Figure S5 (Supporting Information F), proving the contribution of the u-GaN layer to the resistance between the metal contact and the 2DHG. The value of the barrier at the NiAu/p++GaN interface, Φ_1_, is obtained by fitting the simulated *I*–*V* curves with experimental data for all u-GaN thicknesses. The best fitted barrier is Φ_1_ = 0.1 eV. Band diagrams in Figure 4(a) reveal the downward bending of the valence band, due to depletion at the p+GaN/u-GaN interface, resulting in an internal built-in potential, Φ_2_, for thicknesses >5 nm. Evidence for the existence of this barrier is demonstrated in Supplementary Figure S6. At 5 nm, the valence band energy is nearly flat (blue curve in Figure 4(a)). Φ_2_ varies with u-GaN thickness, at the p+GaN/u-GaN interface as shown in Figure 4(b), resulting in a total barrier Φ = Φ_1_ + Φ_2_ = 0.34 eV for 18 nm, as highlighted in the inset of Figure 4(b), matched to experiment via the inclusion of *Q*_it_. The fact that Φ_2_ is twice as large as Φ_1_ at *t*_c_ > 18 nm indicates that the current is controlled by the p+GaN/u-GaN interface rather than the metal/p++GaN contact. Figure 4(b) also illustrates that Φ_2_ saturates as *t*_c_ > 18 nm, due to Fermi pinning at the p+GaN/u-GaN interface, highlighted by the red circle in Figure 4(a). The small value of Φ_1_ and the relative invariance of Φ_2_ at u-GaN thicknesses larger than 18 nm explain why our specific resistances are relatively constant at 5.8 × 10^–4^ Ω cm^2^ and cannot be reduced further by optimizing the stack. This can only be explained by the (*Q*_it_) which induces an upward shift of the VBM at the p+GaN/u-GaN interface (inset of Figure 4(b)), keeping Φ_2_ pinned at 0.25–0.27 eV for *t*_c_ > 18 nm.

**Figure 4:**
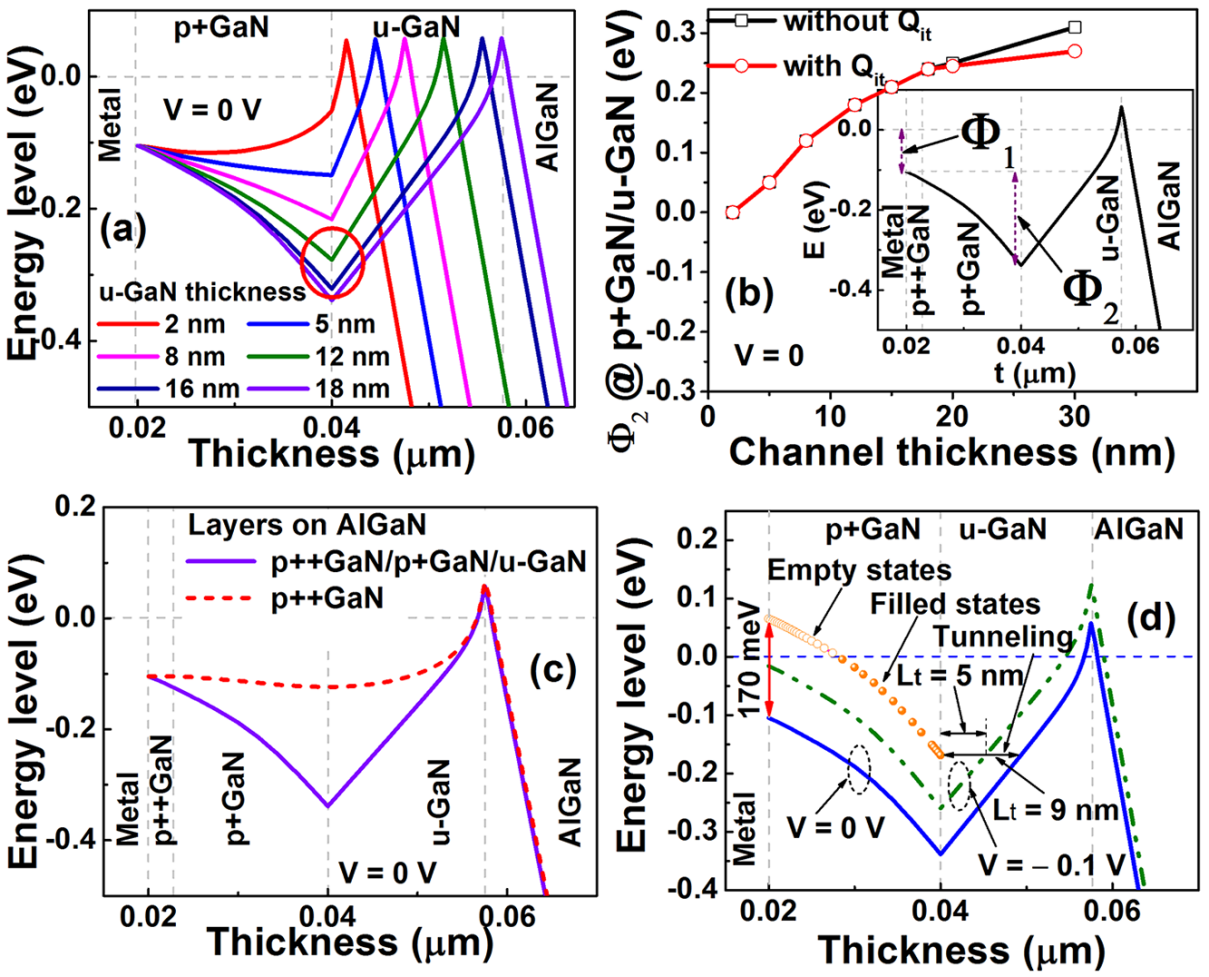
(a) The valence band maximum (VBM) as a function of channel thickness at equilibrium (*V* = 0 V) with interface traps at the p+GaN/u-GaN interface. (b) Built-in potential at the p+GaN/u-GaN interface as a function of channel thickness with and without *Q*_it_. The figure in the inset describes the two barriers present in a NiAu/p++GaN/p+GaN/u-GaN structure. (c) The VBM of p++GaN/AlGaN and p++GaN/p+GaN/u-GaN/AlGaN, when Φ_1_ = 0.1 eV. The formation of Φ_2_ is only observed with p+GaN and u-GaN layers. (d) Schematic based on TCAD simulations to highlight the influence of voltage on the tunneling distance from p+GaN to the 2DHG. Current transport through the p+GaN is via diffusion of holes through acceptors, while that from p+GaN to u-GaN is tunneling at a small applied voltage, which reduces Φ_2_ and tunneling distance *L*_t_ to the 2DHG.

To investigate the causes of the built-in potential barrier Φ_2_, two structures are compared in Figure 4(c): (i) the present p++GaN/p+GaN/u-GaN/AlGaN; and (ii) p++GaN/AlGaN, without the u-GaN layer. In both cases, the Schottky barrier Φ_1_ is assumed to be 0.1 eV. Figure 4(c) indicates that the internal potential Φ_2_ occurs in the presence of u-GaN. This result explains why Chowdhury et al. obtained a smaller contact resistivity of 4.9 × 10^–6^ Ω cm^2^ by using the structure NiAu/p++GaN/AlGaN.^14^ In their structure, Φ_2_ = 0 V, so their specific contact resistivity can be optimized by using a cleaning process to reduce Φ_1_ at the metal/p++GaN (0.1 eV in this study). However, without a u-GaN layer, the devices showed a mobility of 7.5 cm^2^/V s^14^ in comparison to 11 cm^2^/V s in their FINFET device with 20 nm of u-GaN.^3^ This degradation could be due to scattering of carriers at the p++GaN to 2DHG interface.^38,39^ The increase of Φ_2_ with u-GaN thickness in Figure 4(a,b) therefore underlies the increase of sheet resistance in Figure 2. The considerations in separating resistivity at the metal/p++GaN and p+GaN/u-GaN via the TLM method are discussed in Supplementary Figure S7.

Figure 4(d) can be used to differentiate the contributions of current flow through the p+GaN to u-GaN layers. It is seen that at equilibrium (*V* = 0 V), the Fermi level (dashed blue line) crosses the acceptor level, assumed here to be 170 meV from the VBM. Note that acceptor states are empty (open circles in orange) above the Fermi level and filled below, so holes may transport through the p+GaN via diffusion through these acceptor states (Figure 4(d)). At the u-GaN/p+GaN interface, the barrier varies with tunneling distance (*L*_t_) from 5–9 nm, between p+GaN and u-GaN for *V* = −0.1 and 0 V respectively as highlighted. The barrier arising from the acceptor level to the VBM is 170 meV, so at a small applied voltage of −0.1 V (green curve), the current tunnels through this interface. The total current is found to reduce with acceptor level (E_AB_) from 110 to 190 meV (Supplementary Figure S8), corresponding to the position of empty acceptor states in the band gap.

In conclusion, the nature of current transport from a metal contact through a 2DHG is examined. The total current is controlled by two factors, a Schottky barrier at the NiAu/p++GaN contact (0.1 eV in our experiments) and a second barrier of 0–0.25 eV at the p+GaN/u-GaN interface. At a u-GaN thickness less than 5 nm, the u-GaN has no effect on the total current from the metal through to the 2DHG (assuming an absence of dopant scattering at this thickness), though mobility is likely degraded by up to a factor of 3. This is opposite to the case where the u-GaN thickness is larger than 5 nm, where the impact of the barrier at the p+GaN/u-GaN overwhelms that of the barrier at the NiAu/p++GaN interface. The tunneling current through this stack is assisted by empty acceptor states with energy level of 170 meV from the valence band maximum, resulting in a nonideality factor, *n,* between 6–12. This is the first discovery that clearly explains the resistivity increase with u-GaN thickness up to ∼15–20 nm. Also, the best-in-class of Ohmic contacts of resistivity ∼5.0 × 10^–4^ Ω cm^2^, independent of u-GaN thickness from 16 to 30 nm, are demonstrated for the GaN/AlGaN/GaN heterostructure.

## Supporting Information Available

Device fabrication; additional information about the TLM method and sheet resistance; temperature dependent **I*–*V** characteristics; additional information about the Richardson constant; description of the TCAD simulation; dependence of channel thickness on **I*–*V** characteristics; evidence for the presence of the built-in barrier height Φ_2_; reconsideration of the TLM method used to extract resistivity of the metal/p+GaN/u-GaN/AlGaN structure; dependence of acceptor level E_AB_ on **I*–*V** characteristics (PDF)
