# Supplementary material for: Origins of the Schottky Barrier to a 2DHG in a Au/Ni/GaN/AlGaN/GaN Heterostructure
Source: ACS Appl Electron Mater. 2022 Sep 21;4(10):4808–13. doi: 10.1021/acsaelm.2c01138 (PMC9609307; doi:10.1021/acsaelm.2c01138)
Supplement: Supplementary file 1 — el2c01138_si_001.pdf [file el2c01138_si_001.pdf]

Supporting information:

Origins of the Schottky barrier to a 2DHG in an Au/Ni/GaN/AlGaN/GaN Heterostructure

Huy-Binh Do†, Jinggui Zhou †, Maria Merlyne De Souza *, †.

†Department of Electronic and Electrical Engineering, University of Sheffield- North campus, S37HQ, Sheffield, UK.

Corresponding Author

* m.desouza@sheffield.ac.uk.

A. Device fabrication

The GaN wafers were commercially grown by metal organic chemical vapor deposition (MOCVD) on 3″ sapphire substrates. The wafers consisted of 800 nm thick high resistivity u-GaN layer grown on a GaN buffer, followed by 47 nm of $Al_{0.23}Ga_{0.77}N$ layer. Above this, u-GaN layers of thickness 16 nm, 18 nm, 20 nm and 30 nm were selected for this study. The layer above consisted of 17 nm Mg-doped p+ GaN (5×10^{19} cm^{-3}), overlaid by 3 nm of Mg-doped p++ GaN (2×10^{20} cm^{-3}). To investigate the resistivity, rectangular-bar Transmission Line Method (TLM) patterns were fabricated by lift-off with a $W \times L = 200 \times 100\ \mu m^2$, and gap spacing from 5μm to 20 μm. Before deposition of Ni/Au (20 nm/50 nm), the samples were subjected to a two-step surface treatment of BOE and HCl.

B. Additional information about TLM method and sheet resistance

In a typical TLM method, the slope of the total resistance versus gap length gives the sheet resistance per unit width, that represents the resistance inside the semiconductor, the x-intercept is $2L_T$, the transfer length, that represents the distance of current crowding from the edge of the contact (μm), and the y-intercept is $2R_c$, the contact resistance, which is the limit of a zero length resistor within the semiconductor, and is hence a property of the metal-semiconductor interface. The corresponding sheet resistance and contact resistivity, measured at ± 0.5 V and shown in Fig.

2(c), are extracted from the resistance-distance characteristics shown in Fig. 2(b). In other reports, Chen et al. measured R_{sh} and ρ_c at ±5.0 V because an offset voltage around 0 V was observed in his study[1], while Jena et al. measured these values at current of 50 mA/mm[2].

C Temperature dependent IV characteristics

Temperature dependent IV characteristics are conducted in Fig. S1, indicating that a lower temperature leads to higher current at higher voltages, but in the Schottky region (near 0V), unusually, there is barely any change of current with temperature. The results in Fig. S1 are used to calculate ideality factors, Schottky barrier in Fig. 3.

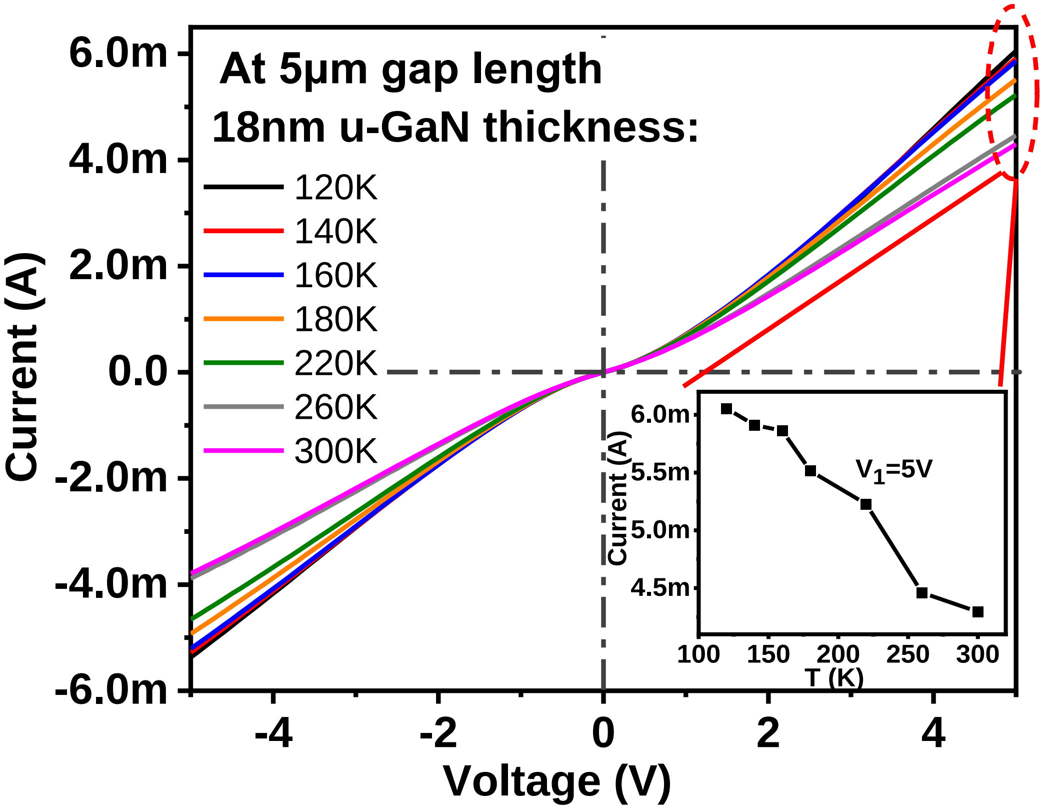

Figure S1. IV characteristics as a function of temperature at a gap of 5μm for 18nm u-GaN thickness. The current increases with reducing temperature due to predominance of the 2DHG in the total resistance. At lower temperature, current increases because of a reduced SB as well as reduced phonon scattering. Inset shows the current versus temperature at 5V.

D. Additional information about Richardson constant

The Richardson constant A^*, defined by $A^* = 4\pi q k^2 m^*/h^3$ can be traditionally obtained by a plot of ln (I/T^2) versus (1/T), but this method results in large uncertainty, in our case, not only because the total current increases at lower temperatures [3], due to the predominance of the 2DHG in the carrier conduction path, but also a large non-ideality factor evident from the I-V characteristics [4]. Previous values of A* have been quoted as 27.9 $A/cm^2/K^2$ by Greco [5], whereas Yao Lin reports 103.8 $A/cm^2/K^2$ [6]. A range of effective masses, m^*have been variously used in analyses of the contact resistivity of p-GaN, viz, $0.81m_0$[5],$1.25m_0$[7], $0.6m_0$[6], (m_0 is the free electron mass). The band structure of GaN consists of a heavy hole, light hole and split off hole but it is the effective mass of the split-off holes, which despite their low Fermi-Dirac occupation factors, plays the most important influence on the tunneling flux of a GaN contact, whereas the direction of current flow in the 2DHG is in the transverse direction [8]. Santic reports an average value of the tunneling mass as 0.16 summarized in his review, in agreement with a value of 0.2 from more recent theoretical work by Carvalho et al [9].

E. Description of TCAD simulation

In our simulation, the Mg profile of 3.0e19/cm^3 for p^{++} GaN is incorporated as shown in Fig. S2(a), an order of magnitude lower concentration of 3.0e18/cm^2 is assumed in the p^+ GaN, and 5.6e17/cm^3 in the u-GaN layer according to a SIMS profile[10]. The activation energy of Mg is assumed to be 170 meV as a best fit to our experimental data across the range of temperatures[11] although it has been variously reported between 112 eV to 190 eV[11] depending upon concentration. It generally decreases as the Mg-concentration increases, however, when the Mg-concentration is of the order of 1e21 cm^{-3}, the activation energy increases again[11]. Trap kinetics are described by Shockley-Read-Hall, incomplete ionization and Auger recombination, whereas mobility is described by the Albrecht model for low field and the nitride specific field dependent model for high field, adjusted to give a maximum value of 15 cm^2/Vs[12]. The Universal Schottky tunneling (UST) model and Poole-Frenkel Barrier Lowering for Coulombic wells are used to simulate a tunneling current at the NiAu/p++GaN interface. Fig. S2(b) shows the behavior of **μ** (experiment versus model) between 180K-300K, in good agreement with the reported values from experiment[3]

and model. The TCAD models deviate outside this range, due to incorrect capture of phonon scattering of holes in GaN. The resultant IV characteristics in Figure S4 fit well with experiment at all channel thicknesses (16 nm, 18 nm, 20 nm, and 30 nm) when interface traps (Q_{it}) of $1 \times 10^{17} cm^{-2}$, with the energy level located close to $E_v + 0.6\ eV$, are included at the p+GaN/u-GaN interface as per the inset of Fig. S4.

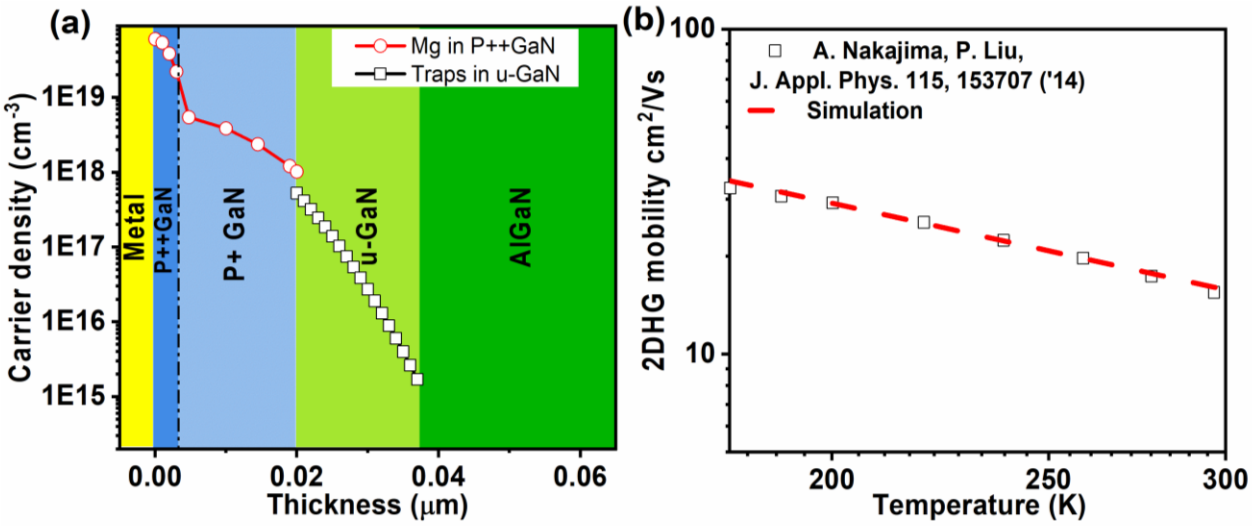

Figure S2. (a) Mg doping profile implemented in simulations. (b) TCAD simulation of mobility as a function of temperature, benchmarked against experiment from A.Nakajima, et. al[3].It is reported that hole traps with energy levels of 0.46 – 1.3 eV above E_V occupying in lightly Mg-doped GaN[13], so the effects of hole traps at p+GaN/u-GaN interface on current-voltage characteristic were investigated. The best fitted-level of hole trap is at 0.6 eV above E_V with the concentration of 1e17 cm-3, corresponding to reported carbon contamination of 3e16 to 6e17 cm-3 in GaN[14]. The experimental and simulated results are fitted well in Fig. S4. Without hole traps, current shows more Schottky behavior in Fig. S3

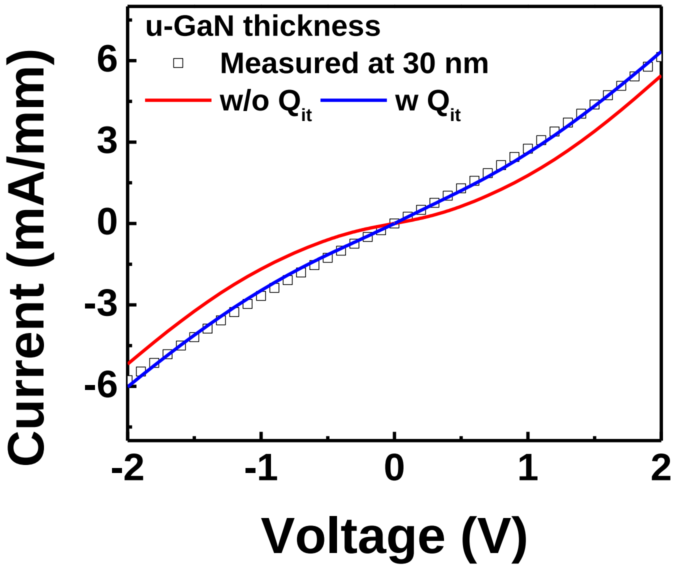

Figure S3. The simulated and experimental IV characteristics with and without interface traps at p+GaN/u-GaN interface.

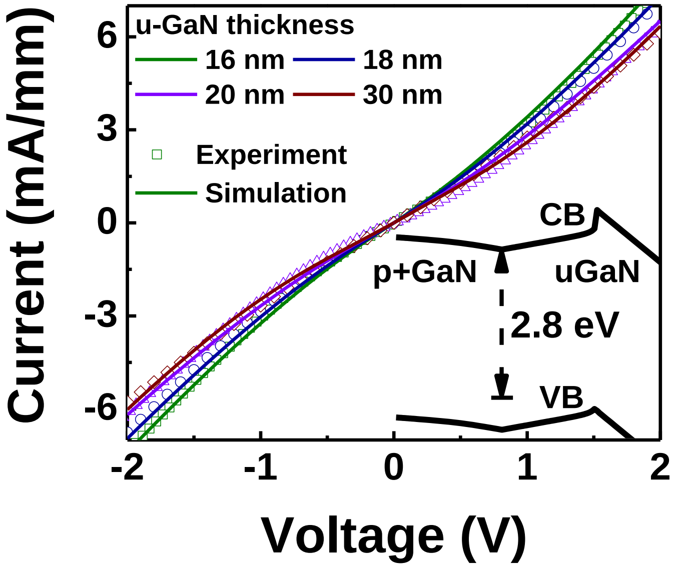

Figure S4. Simulated and experimental IV characteristics for different thicknesses of the u-GaN layer, all figures are based on the measured resistivity and Schottky barrier (SB) at 300K with 5 μm distance between electrodes. The figure in the inset represents an acceptor trap.

F. Dependence of channel thickness on IV characteristics

The effects of channel thickness on the resistivity of metal/2DHG is investigated in Fig. S5. It is shown that Ohmic behavior is observed when u-GaN thickness is less than 5 nm, while Schottky behavior determines the current transportat mechanism at larger u-GaN thickness. The result indicates that u-GaN thickness have a key-role in reducing total resistance of metal/2DHG contact.

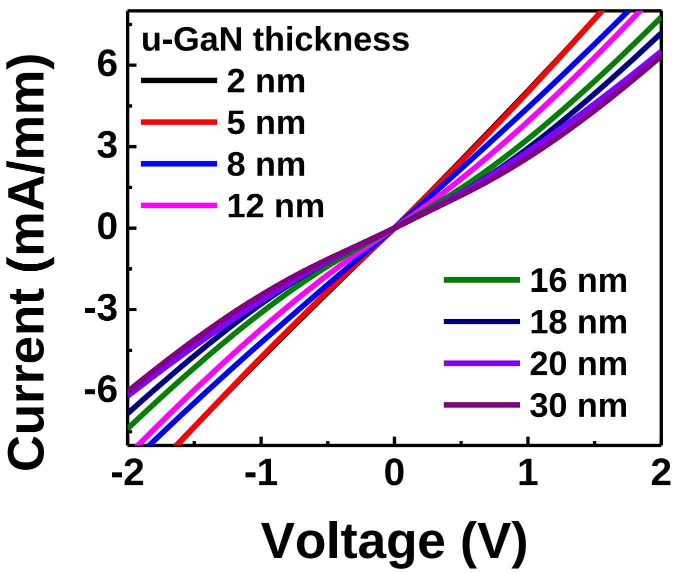

Figure S5: The simulated IV characteristics as a function of channel thickness. The linear behavior is observed when channel thickness decreases.

G. Evidence for the presence of the built-in barrier height Φ_2

An ideal Ohmic contact at the metal/p++GaN interface is simulated in Fig. S6. The Schottky behavior is observed even when Φ_1 = 0 eV for t_c = 18 nm. This Schottky behavior is due to the presence of a built-in barrier height Φ_2. The effect of assuming an Ohmic contact (Φ_1 = 0 eV) is also observed for t_c = 2 nm when Φ_2 = 0 eV shown in Fig. S6.

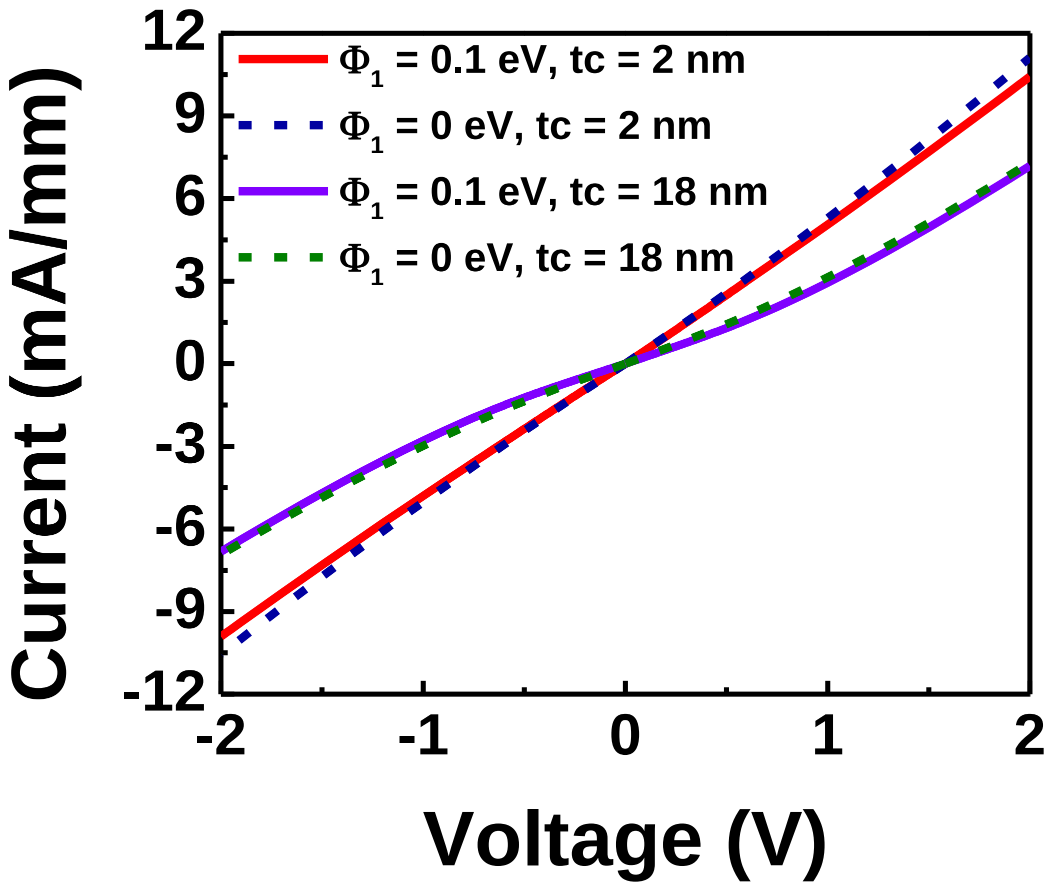

Figure S6: The simulated IV characteristics when $\Phi_1 = 0$ V and $\Phi_1 = 0.1$ eV for u-GaN thicknesses of 2 nm and 18 nm.

H. Reconsideration of the TLM method used to extract resistivity of the metal/p+GaN/u-GaN/AlGaN structure

A constant value of resistivity at the NiAu/p++GaN contact and the existence of the second Schottky barrier at p+GaN/u-GaN interface enables reconsideration of the TLM method in extracting a contact resistivity. In a normal metal/semiconductor structure, the measured total resistance can be expressed by equation (S1):

$R_T = 2R_m + 2R_C + R_{semi}$, (S1)

where R_m is the resistance due to the contact metal, R_C is associated with the metal/semiconductor interface, and R_{semi} is the semiconductor resistance. In most cases, $R_C >> R_m$, so R_m can be ignored.

In a metal/2DHG contact, the total resistance illustrated in Fig. S8, can be calculated using equation (S2).

$$R_T = 2R_C + 2R_{u-GaN/p+GaN} + R_{semi}, \text{(S2)}$$

where $R_{u\text{-}GaN/p+GaN}$ is the resistance due to the Schottky barrier at u-GaN/p+GaN interface, and $R_{u\text{-}GaN/p+GaN} = 0$ when tc = 0. $R_{semi} = 2R_{sh1} + 2R_{sh2} + R_{shH}$ when $t_c \neq 0$, and $R_{semi} = 2R_{sh1} + R_{shH}$ when $t_c = 0$. In these formulas, R_{sh1}, R_{sh2}, R_{shH} are factors contributing to the sheet resistance in a p++GaN region, a u-GaN region, and 2DHG, respectively. Fig. S8 can be used to explain the fact that sheet resistance increases as t_c increases in Fig. 2(c), due to the increase of R_{sh2}. In a conventional metal/semiconductor structure, the intercept of R_T – gap length with R_T gives the value of $2R_C$, however, this value should be $2R_C + 2R_{u\text{-}GaN/p+GaN}$ in a metal/2DHG contact. Equation (S2) illustrates a strong dependence of resistivity on channel thickness extracted by the TLM method. It also explains why extracted resistivity is low at small t_c or at $t_c = 0$, while that at large t_c has high resistivity and cannot be reduced in the Table of Content (TOC) figure because we cannot separate R_C and $R_{u\text{-}GaN/p+GaN}$ via the TLM method.

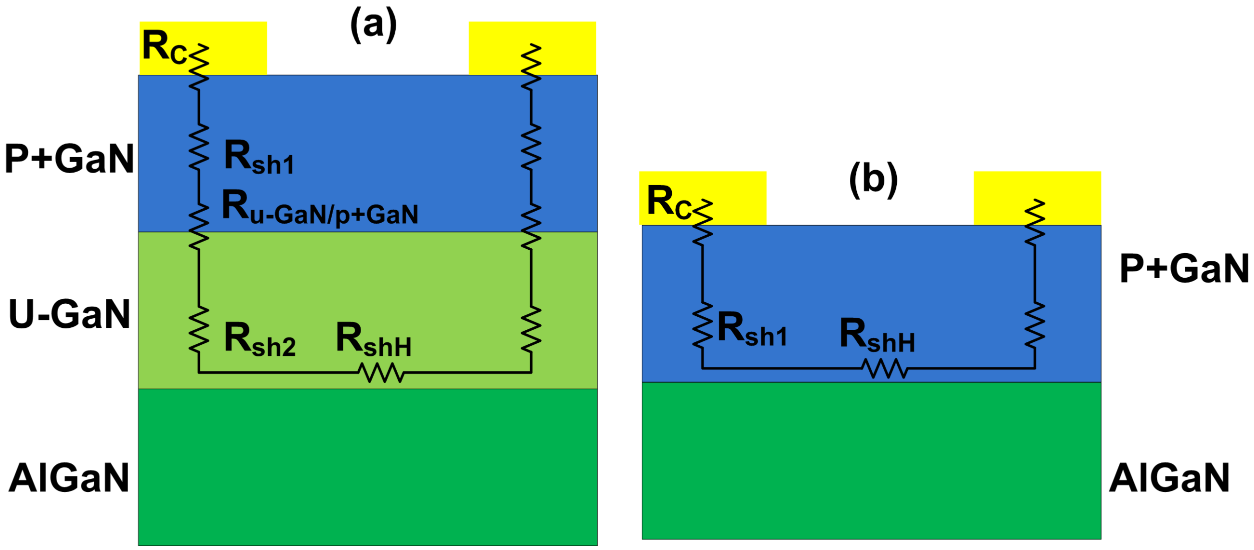

Figure S7. Cross-section of the substrate and TLM contacts in Ni/Au, highlighting the contributions of the various layers in the structure.

I. Dependence of the acceptor level, E_{AB} ,on IV characteristics

Figure S7 indicates that the acceptor level E_{AB} significantly affects the contact resistance between the metal and the 2DHG. The larger value of E_{AB} results in an increase in the bending of IV curves around 0 V, due to an increase of barrier between the acceptors states in p++ GaN from the Valence Band Maximum (VBM) (See Fig 4d of manuscript).

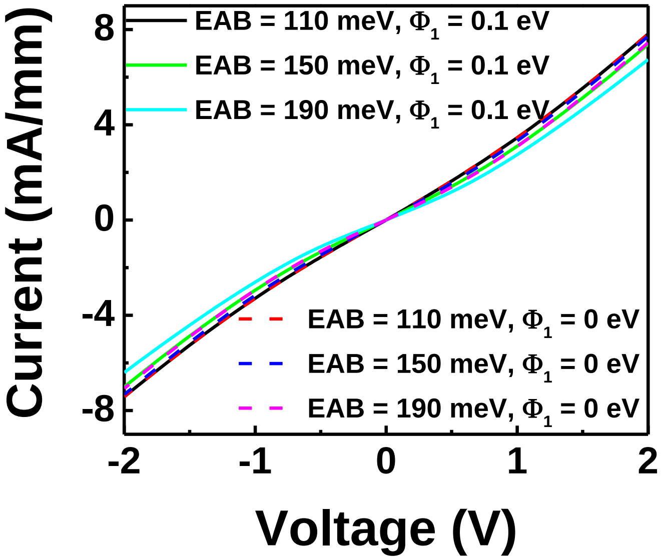

Figure S8. Dependence of acceptor level on the current. Schottky barrier is observed even when Φ_1 = 0 eV at a higher E_A value of 190 meV.

References

(1) Zheng, Z.; Song, W.; Zhang, L.; Yang, S.; Wei, J.; Chen, K. J. High ION and ION/IOFF Ratio Enhancement-Mode Buried p-Channel GaN MOSFETs on p-GaN Gate Power HEMT Platform. *IEEE Electron Device Lett.* **2020**, *41* (1), 26–29. https://doi.org/10.1109/LED.2019.2954035.

(2) Bader, S. J.; Chaudhuri, R.; Hickman, A.; Nomoto, K.; Bharadwaj, S.; Then, H. W.; Xing, H. G.; Jena, D. GaN/AlN Schottky-Gate p-Channel HFETs with InGaN Contacts and 100 MA/Mm on-Current. In *2019 IEEE International Electron Devices Meeting (IEDM)*; IEEE, 2019; Vol. 51, pp 4.5.1-4.5.4. https://doi.org/10.1109/IEDM19573.2019.8993532.

(3) Nakajima, A.; Liu, P.; Ogura, M.; Makino, T.; Nishizawa, S. I.; Yamasaki, S.; Ohashi, H.; Kakushima, K.; Iwai, H. Temperature-Independent Two-Dimensional Hole Gas Confined at GaN/AlGaN Heterointerface. *Appl. Phys. Express* **2013**. https://doi.org/10.7567/APEX.6.091002.

(4) Schroder, D. K. *Semiconductor Material and Device Characterization*; John Wiley & Sons, Inc.: Hoboken, NJ, USA,

2005; Vol. 18. https://doi.org/10.1002/0471749095.

(5) Greco, G.; Prystawko, P.; Leszczyski, M.; Lo Nigro, R.; Raineri, V.; Roccaforte, F. Electro-Structural Evolution and Schottky Barrier Height in Annealed Au/Ni Contacts onto p-GaN. *J. Appl. Phys.* **2011**, *110* (12). https://doi.org/10.1063/1.3669407.

(6) Lin, Y. J. Application of the Thermionic Field Emission Model in the Study of a Schottky Barrier of Ni on P-GaN from Current-Voltage Measurements. *Appl. Phys. Lett.* **2005**, *86* (12), 1–3. https://doi.org/10.1063/1.1890476.

(7) Okumura, H.; Martin, D.; Grandjean, N. Low p -Type Contact Resistance by Field-Emission Tunneling in Highly Mg-Doped GaN. *Appl. Phys. Lett.* **2016**, *109* (25). https://doi.org/10.1063/1.4972408.

(8) Santic, B. On the Hole Effective Mass and the Free Hole Statistics in Wurtzite GaN. *Semicond. Sci. Technol.* **2003**, *18* (4), 219–224. https://doi.org/10.1088/0268-1242/18/4/305.

(9) De Carvalho, L. C.; Schleife, A.; Bechstedt, F. Influence of Exchange and Correlation on Structural and Electronic Properties of AlN, GaN, and InN Polytypes. *Phys. Rev. B - Condens. Matter Mater. Phys.* **2011**, *84* (19). https://doi.org/10.1103/PhysRevB.84.195105.

(10) Nakajima, A.; Liu, P.; Ogura, M.; Makino, T.; Kakushima, K.; Nishizawa, S.; Ohashi, H.; Yamasaki, S.; Iwai, H. Generation and Transportation Mechanisms for Two-Dimensional Hole Gases in GaN/AlGaN/GaN Double Heterostructures. *J. Appl. Phys.* **2014**, *115* (15), 153707. https://doi.org/10.1063/1.4872242.

(11) Kozodoy, P.; Xing, H.; DenBaars, S. P.; Mishra, U. K.; Saxler, A.; Perrin, R.; Elhamri, S.; Mitchel, W. C. Heavy Doping Effects in Mg-Doped GaN. *J. Appl. Phys.* **2000**, *87* (4), 1832–1835. https://doi.org/10.1063/1.372098.

(12) Kumar, A.; De Souza, M. M. Impact of Channel Thickness on the Performance of an E-Mode p-Channel MOSHFET in GaN. *Appl. Phys. Lett.* **2018**, *112* (15). https://doi.org/10.1063/1.5021306.

(13) Narita, T.; Tokuda, Y.; Kogiso, T.; Tomita, K.; Kachi, T.. The Trap States in Lightly Mg-Doped GaN Grown by MOVPE on a Freestanding GaN Substrate. *J. Appl. Phys.* **2018**, *123* (16), 161405. https://doi.org/10.1063/1.5010849.

(14) Seager, C. H.; Tallant, D. R.; Yu, J.; Götz, W. Luminescence in GaN Co-Doped with Carbon and Silicon. *J. Lumin.* **2004**, *106* (2), 115–124. https://doi.org/https://doi.org/10.1016/j.jlumin.2003.08.004.
